# Editorial: Special Issue “Laser-Generated Periodic Nanostructures”

**DOI:** 10.3390/nano11082054

**Published:** 2021-08-12

**Authors:** Peter Simon, Jürgen Ihlemann, Jörn Bonse

**Affiliations:** 1 Institut für Nanophotonik Göttingen e.V., Hans-Adolf-Krebs-Weg 1, D-37077 Göttingen, Germany; 2 Bundesanstalt für Materialforschung und -prüfung (BAM), Unter den Eichen 87, D-12205 Berlin, Germany

The study of laser-fabricated periodic nanostructures is one of the leading topics of today’s photonics research. Such structures on the surface of metals, semiconductors, dielectrics, or polymers can generate new material properties with special functionalities. Depending on the specific material parameters and the morphology of the structures, new devices such as microlasers, optical nanoswitches, optical storage devices, sensors or antifraud features can be realized. Furthermore, laser-generated surface textures can be used to improve the tribological properties of surfaces in contact and in relative motion—to reduce friction losses or wear [1,2,3], to modify the wettability or the cell and biofilm growth properties of surfaces through bioinspired laser engineering [4], for emerging medical applications [5,6], or as decoration elements for the refinement of precious goods [7].

This Special Issue “Laser-Generated Periodic Nanostructures” focuses on the latest experimental and theoretical developments and practical applications of laser-generated periodic structures that can be generated in a “self-organized” way (laser-induced periodic surface structures, LIPSS, ripples) [8,9,10,11] or via laser interference-based direct ablation (often referred to as direct laser interference patterning, DLIP) [12,13,14]. We aimed to attract both academic and industrial researchers in order to collate the current knowledge of nanomaterials and to present new ideas for future applications and new technologies. By 8 August 2021, 22 scientific articles have been published in the Special Issue [15,16,17,18,19,20,21,22,23,24,25,26,27,28,29,30,31,32,33,34,35,36], see www.mdpi.com/journal/nanomaterials/special_issues/laser-generated_periodic.

Three “Feature Papers” [15,16,17] were invited by the Guest Editors for the adequate framing of this Special Issue. An additional 19 papers were published as regular contributions [18,19,20,21,22,23,24,25,26,27,28,29,30,31,32,33,34,35,36]. The publications in this Special Issue can generally be divided into three groups: (1) manuscripts related to self-organized laser-induced periodic surface structures [15,18,19,21,23,24,25,26,28,30,31,32,33,34,35,36], (2) manuscripts related to interference-based periodic surface structuring [17,22,27,29], or (3) manuscripts combining both approaches [16,20].

Several publications focus on the relevance of chemical surface alterations during or after laser-processing, including studies of superficial oxidation on the formation of specific types of LIPSS [18,23,24,25,30], chemo-capillary effects in DLIP [29], or even post-laser irradiation effects due to reactions with the environment [30]. Other publications study the fundamental mechanisms of structure formation on the basis of experimental and theoretical plasmonic approaches [15,21,26], and also in combination with a two-temperature model [28], advanced molecular dynamics simulations [22], or multi-physical hydrodynamic continuum considerations [31,34]. Two articles investigate in vitro the growth of different types of biological cells (either scar-forming fibroblasts or bone-forming osteoblasts) on laser-generated micro- and nanostructures for medical applications [19,25]. Other applications qualify the real-life anti-icing properties of large-area laser-structured surfaces for aeronautic applications [17], and the alteration of surface superconductivity is demonstrated with a view toward future energy applications [24].

In the following, all publications in this Special Issue are briefly summarized, ordered by their date of publication.

Florian et al. [18] reported on a strong absorbing, oxidation-prone material (chromium nitride, CrN), an unusual type of self-organized near-wavelength-sized nanostructure—so-called low spatial frequency LIPSS (LSFL-‖), being parallel to the femtosecond laser (790 nm, 30 fs, 1 kHz, or 1030 nm, 350 fs, 100 kHz) beam polarization used for irradiation in air. This is in contrast to the common LSFL (LSFL-⟂) that usually form perpendicular to the laser beam polarization on strong absorbing materials. Numerical simulations of the intrasample optical beam propagation by means of finite-difference time-domain (FTDT) calculations revealed the scattering rough oxide layer surface along with an ~100 nm thick oxide layer as the origin of the LSFL-‖ being “imprinted” at the buried oxide/sample interface.

Klos et al. [19] created tailored surface micropatterns on titanium alloy (Ti6Al4V) that were covered by different multiscale surface morphologies (nanometric LIPSS (LSFL) or hierarchical micro/nanospikes) upon fs-laser (1030 nm, 400 fs, 10 kHz or 100 kHz) scan-processing in air. The authors observed that human mesenchymal stem cells (hMSCs) can be spatially controlled and mechanically strained by these laser-processed topographies. Additionally, the surface nanostructures affected the surface wettability and adsorption of proteins. As consequence, the focal cell adhesion was influenced and finally induced surface shape-related mechanical constraints on cells that can specifically promote osteogenic differentiation.

Mezera et al. [20] employed a two-step approach for creating hierarchical micro/nanostructures on polycarbonate polymer surfaces by combining DLIP—using a UV ns-laser (266 nm, 3 ns, 2 kHz) as the first step—with the subsequent processing of LIPSS using a UV ps-laser (350 nm, 7 to 10 ps, 100 kHz) as the second step. An experimental focus was laid on the influence of the direction of the laser beam polarization on the resulting surface structures, and was theoretically complemented by finite-difference time-domain (FDTD) calculations. Attenuated total reflection Fourier-transform infrared spectroscopy (ATR-FTIR) and micro-Raman spectroscopy (µ-RS) provided additional information on the structural and chemical polymer alteration upon laser-processing, revealing an enhanced polymer degradation for increasing accumulated fluence levels.

Kunz et al. [15] demonstrated in their Feature Paper that an additional gold layer, less than 300 nm thin, deposited on a wide band gap dielectric (fused silica) can significantly improve the regularity and reproducibility of so-called high spatial frequency LIPSS (HSFL) on the dielectric during large-area ultrashort laser scan-processing (1025 nm, 300 fs, 100 kHz) in air. Simultaneously, an increased total transmission of the silica glass samples, accompanied with a change in the surface wetting behavior, was proven. The beneficial effect of the Au-layer is attributed to a more homogeneous coupling of the electromagnetic laser radiation to the sample material, which reduces the influence of material properties, nonlinearities, and laser energy fluctuations. Moreover, the highly reflective gold film may act as a protective layer for those areas of the fluence profile that are insufficient for HSFL formation.

Takaya et al. [21] irradiated silicon suboxide (SiO_x_, x ≈ 1) with femtosecond laser pulses (800 nm, 100 fs, 10 Hz) in air. Upon multipulse irradiation at moderate fluences around 700 mJ/cm^2^, the formation of periodic nanogratings with a period in the range of 200–300 nm was observed. The laser-induced formation of a thin surface layer with a high electron density, followed by nanoablation by plasmonic near-fields, were identified to lead to the observed patterns. Silicon suboxide is a material of high interest as it can be transferred to silica glass by thermal oxidation; the proposed process provides a useful approach for the rapid generation of nanostructures on glass.

Blumenstein et al. [22] presented a direct comparison of experimental and theory-based simulation results of UV laser-induced surface nanostructuring of gold. They investigated the structure formation after ultrashort pulse laser (248 nm, 1.6 ps) irradiation. The gold surface was exposed to a spatially periodic intensity profile with a sinusoidal shape and periods of 270 nm, 350 nm, and 500 nm (two-beam interference). For the simulations on an identical spatial scale, a hybrid atomistic-continuum model (molecular dynamics two-temperature model, MD-TTM) was used to model the interaction of the laser pulse with the gold target and the subsequent time evolution of the system. An excellent agreement between the modeling results and experimental data was found; especially the formation of narrow nanoridges—composed of two colliding side walls of neighboring grooves—was found in the simulation as well as in the experiment.

Sinev et al. [23] demonstrated the formation of oxidative LIPSS on ~10 nm thick metallic titanium films on BK7 glass upon scan-processing with an IR ns-fiber laser (1070 nm, 4–50 ns, 10–100 kHz). Regular LSFL with periods around 720 nm, modulation depths between 70 nm and 110 nm, and an orientation perpendicular to the linear laser beam polarization were characterized by optical and atomic force microscopy (AFM). According to the authors’ complementary calculations, the laser-induced peak temperatures stayed below the melting temperature of the titanium for the chosen irradiation conditions.

Cubero et al. [24] provided the first experimental proof that the surface superconductive properties (e.g., the surface critical field strengths *H*_c3_) of 25 µm thick niobium foils can be controlled through fs-laser generated LSFL featuring spatial periods between 570 nm and 775 nm, modulation depths around 200 nm, and an orientation perpendicular to the linear laser beam polarization (790 nm, 30 fs, 1 kHz, or 1030 nm, 280 fs, 1 kHz; irradiation in air, nitrogen, or argon). Moreover, a clear correlation was found between the relative orientation of the magnetic field and the periodic surface patterns.

Petrović et al. [25] studied the response of NIH 3T3 fibroblast cells on LIPSS (LSFL) processed with a Yb:KGW-laser system (1030 nm, 160 fs, 1 kHz) on a multilayer system consisting of 15 (Ti/Zr)-bilayers on a silicon substrate. The micro/nanopatterns of altered chemical/structural composition (qualified by energy dispersive X-ray spectroscopy (EDX), X-ray photoelectron spectroscopy (XPS), depth-profiling Auger electron spectroscopy (AES), scanning electron microscopy (SEM), and transmission electron microscopy (TEM)) were characterized regarding the influences on the cell morphology and cell proliferation and with respect to the possibility of cell orientation along the LSFL ridges. The authors reported that the Ti/Zr-multilayer thin films supported a high degree of cell proliferation and oriented cell growth and should be suitable for implants.

Liu et al. [26] investigated the formation of LIPSS (LSFL) on tilted metal surfaces (steel, titanium, aluminum, copper) upon line scanning irradiation with s-polarized fiber laser pulses (1030 nm or 515 nm, 420 fs, 100 kHz). The authors identified a characteristically different LIPSS orientation between the group of steel and titanium, where the LSFL patterns moderately rotated by some tens of degrees at elevated angles of incidences (*θ* = 30° or 45°), and the other materials Al and Cu, where the LSFL stayed always perpendicular to the laser beam polarization at all angles of incidence (*θ* = 0°, 30°, 45°). The authors proposed that this finding is based on material-specific differences in the electron–phonon coupling factor, which should indirectly affect the propagation length of surface plasmon polaritons (SPPs) involved in the formation of the LSFL.

Soldera et al. [16] presented in their Feature Paper the two-beam DLIP structuring of soda lime glass with visible ps-laser pulses (532 nm, 12 ps or 70 ps, 10 kHz or 1 kHz). Periodic spot- and line-like surface textures were processed featuring spatial distances between 2.3 and 9.0 µm along with depth-to-diameter aspect ratios up to 0.29. Additionally, within these microstructures, LIPSS with spatial periods of ~300 nm were present—resulting in hierarchical micro/nanostructures. The line-patterned samples with periods between 2.3 and 3.9 µm diffracted the white light of a tungsten lamp with efficiencies of approximately 30% into specific diffraction modes. Simultaneously, all laser-processed samples altered their surface wetting behavior with water. In some cases, even the super-hydrophilic state with contact angles CA < 5° manifested.

In another Feature Paper, Milles et al. [17] studied the icephobic performance of hierarchical multiscale laser-textured aluminum alloy (AL2024) surfaces for aeronautic applications. Using ps- and ns-laser sources, the authors employed direct laser writing (DLW) and direct laser interference patterning (DLIP) to process different periodic surface topographies with feature sizes ranging between 2.6 and 50 µm. Some samples were additionally spray-coated using a perfluoropolyether (PFPE) monolayer to enforce super-hydrophobicity. Subsequently, the authors investigated the adhesion strength of ice on the microtextured surfaces. Moreover, the icephobic performance was tested in an icing wind tunnel, simulating real-world icing conditions. The authors demonstrated that optimized surface textures, consisting of cross-like DLIP patterns with a size of 2.6 µm, can lead to a relative reduction of the ice adhesion strength by ~90% when compared with a polished aluminum alloy surface. The authors proposed that specific types of surface microstructures, i.e., line-like and cross-like topographies, and feature sizes smaller than 5 µm are especially suitable for creating icephobic surfaces.

Nakata et al. [27] made use of complex multi laser beam interference to establish a laser-induced dot transfer technique (a variant of the laser-induced forward transfer, LIFT) that is able to generate periodic arrangements of several-hundred-nanometer-large nanodots on a receiving substrate via a solid–liquid–solid mechanism. Employing a single fs-laser pulse (785 nm, 240 fs) to a platinum donor film on a glass substrate in direct contact with an acceptor substrate in a vacuum chamber setup at a pressure <1.3 kPa, regular and periodic arrangements of almost spherical platinum sub-micrometer-sized nanodots were transferred onto a gold-coated silica glass acceptor substrate.

Kuznietsov et al. [28] manufactured metallic Ti-Fe multilayers, consisting of 15 (Ti/Fe)-bilayers on a steel substrate, with total stack thicknesses between 60 and 80 µm and studied the formation of LIPSS (LSFL) on these coatings upon irradiation with fs-laser pulses (1030 nm, 213 fs, 600 kHz) in a scan-processing approach. The LSFL-covered surfaces were characterized by SEM, XPS, and X-ray diffraction (XRD). Special attention was drawn to the depth-resolved laser-induced modification by using scanning electron microscopy on a previously prepared cross-sectional cut with a focused ion beam (FIB), and to the formation of the intermetallic compound TiFe. The resulting thermal laser-induced effects were simulated by a 1D two-temperature model (TTM) to predict the extent of the heat-affected region, as well as to predict the period of the LIPSS patterns.

Jähnig et al. [29] explored the application of two-beam DLIP for the structuring of steel surfaces containing different amounts of the surface-active element sulfur—allowing them to influence the laser-induced melt flow patterns. Employing single ns-laser pulses (1053 nm, 12 ns), this included the variation of the sulfur content in steel (30 ppm, 100 ppm, ~300 ppm, or 1500–3000 ppm) and the laser fluence. The authors demonstrated that, via thermocapillary melt flows (Marangoni melt convection), single-surface peak geometries with amplitudes up to ~50 nm can be generated for steel with sulfur content below 300 ppm, whereas split-peak topographies were formed for higher sulfur content steel or at high laser fluences. The experiments were complemented by numerical simulations on the basis of a smoothed particle hydrodynamics (SPH) model elucidating the influence of the sulfur content in steel on the melt pool convection during single-pulse ns-DLIP.

Wood et al. [30] systematically explored the influence of the post-laser-processing environment (CO_2_ atmosphere, boiling water) on the water wettability of LIPSS(LSFL)-covered stainless steel (AISI 316) surfaces that were previously scan-processed by fs-laser irradiation (800 nm, <100 fs, 1 kHz) in air. It was found that exposure to a CO_2_-rich environment after micromachining leads to an increased surface hydrophobicity, while residence in a boiling water bath instead leads to a hydrophilic surface. Moreover, important conclusions were drawn regarding the post-laser irradiation surface cleaning for removing nonsintered loosely attached nanoparticles and agglomerates from the surface; if not removed, these nanoparticle agglomerates become hydrophobic, creating a Cassie–Baxter air-trapping layer on the surface that may feature water contact angles up to 180°.

Nakhoul et al. [31] employed sequences of multiple cross-polarized double-fs-laser pulses (800 nm, 25 fs, 1 kHz) to generate a plethora of nanostructures at the surface of polished single-crystalline <100> nickel surfaces, including random patterns with concentrated nanoreliefs, 1D nanostripes, or 2D hexagonal arrays, forming disordered, labyrinthine, and bumpy nanopatterns. The surface morphologies were characterized by SEM and AFM. Some of the nanostructures featured diameters of only a few tens of nanometers, and heights up to 100 nm. The authors demonstrated that the surface structures can be controlled by the specific laser irradiation conditions, i.e., particularly the laser fluence and number of irradiating double-pulse sequences. Different ablation phenomena and hydrodynamic instabilities were discussed as potential mechanisms driving the formation of the surface nanostructures.

Shavdina et al. [32] studied the spot- and scan-processing of ~180 nm thick polystyrene (PS) films on silicon substrates upon UV fs-laser (266 nm, 100 fs, 50 Hz or 1 kHz) irradiation in air using large numbers of effective pulses per beam spot area ranging between 10^3^ and 10^7^. The regimes for optimum LIPSS formation were systematically explored and the influence of the thermal heating was tested for different substrate temperatures below the materials’ softening point (*T*_g_), revealing an increase in the LIPSS modulation depth from ~12 nm (RT) to ~20 nm (97 °C).

Dominic et al. [33] investigated the formation of subwavelength high spatial fr quency LIPSS (HSFL) on a tungsten surface upon fs-laser irradiation (800 nm, 60 fs, 1 kHz) in different oxidizing or inert environments (air, nitrogen, argon, 10^−7^ mbar high vacuum) and in combination with or without Ar-ion sputter removal of covering oxides prior to the laser irradiation. Their work confirms that surface oxidation is not a prerequisite for HSFL formation on tungsten.

Prudent et al. [34] experimentally studied the fluence- and pulse number-dependent evolution of surface morphology and the influence of interpulse feedback effects on the formation of LIPSS (LSFL and HSFL) on thin metallic glass (Zr_65_Cu_35_ alloy) films on silicon—starting with different film-specific nanosized precursor structures (referred to as coarse and tight columns) and subsequently irradiating them with multiple (up to 50) sequences of single or parallel-polarized double-fs laser pulses (800 nm, 60 fs) in the interpulse delay range up to 70 ps. The authors suggest that the high viscosity of an amorphous alloy can be bypassed through double-fs-pulse irradiation that drives the material towards higher temperatures and lower viscosities—a state that is more favorable to it undergoing capillary melt flows. As a consequence, the temporally tailored deposition of the optical energy allows promotion of the development of homogeneous and regular HSFL, while widely preserving the surface integrity.

Gutiérrez-Fernández et al. [35] used an experimental approach combining laser pulses (8 ns, 10 Hz) at different wavelengths (266 nm, 532 nm) and sequential irradiation with different polarization orientations to generate different nanostructure topographies, in particular linear gratings, two-dimensional grids, and arrays of nanodots on 130 nm thick films of semiconducting polymer poly(3-hexylthiophene) (P3HT) and 20 nm thick films of ferroelectric copolymer poly[vinylidenefluoride-co-trifluoroethylene] (P(VDF-TrFE)), both spin-coated on silicon wafers. Grazing incidence small- and wide-angle X-ray scattering (GISAXS/GIWAXS) performed at a synchrotron radiation beamline allowed them to qualify the crystallinity of the polymeric material in the irradiated regions. Sub-micrometric 2D periodic ferroelectric nanodot structures were laser-processed on a functional polymer bilayer consisting of P(VDF-TrFE) (top)/P3HT (bottom). Its ferroelectric nature was proven through piezoresponse force microscopy (PFM).

Werner and Chowdhury [36] investigated the formation of LIPSS on single-crystalline <111> and <100> silicon wafer surfaces following irradiation with multiple (8 to 10,000) mid-infrared fs-laser pulses (3.6 µm, 200 fs, 500 kHz). The irradiated surface spots were characterized by SEM, AFM, EDX, cross-sectional TEM, and electron beam diffraction (EBD). Extreme subwavelength structures with 50–100 nm sized features arranged parallel to the linear laser beam polarization direction and with a quasi-periodicity of 700 nm were reported. However, the size of the individual surface features depended strongly on the distance from the center of the irradiated spot and approached values down to ~λ/180 nm only. Moreover, TEM revealed that characteristic outgrowing nanospheroidal clusters (NSC) were quenched in an amorphous state from the laser-induced superficial melt/amorphous layer (40 nm).

Four items of this Special Issue “Laser-Generated Periodic Nanostructures” [18,31,33,35] were selected as “Editor’s Choice” articles by the Editorial Office and the international board of Editors of Nanomaterials—based on readers’ interests, academic editors’ comments, and reviewers’ suggestions. It is supposed that such articles will be particularly interesting or important, while simultaneously aiming to provide a snapshot of some of the work published in the various research areas of the journal.

Finally, the Guest Editors would like to express their sincere gratitude to all authors and reviewers of this Special Issue for their intense efforts and to the editorial staff of Nanomaterials for their professional support and guidance.

## Data Availability

Data sharing not applicable as no datasets were generated or analyzed in the current article.
